# Effectiveness of outdoor fitness equipment intervention on health outcomes: a systematic review and meta-analysis

**DOI:** 10.3389/fpubh.2026.1701136

**Published:** 2026-02-23

**Authors:** Zhi-Yuan Tang, Yu-Qin Ji, Yi-Su Zhu, Hong-Bin Xiang, Qiang Ye

**Affiliations:** 1 Department of Basic Education, Nanjing Audit University Jinshen College, Nanjing, China; 2 School of Sport Science, Beijing Sport University, Beijing, China; 3 Graduate School, Nanjing Sport Institute, Nanjing, China; 4 School of Physical Education, Shanghai University of Sport, Shanghai, China; 5 School of Physical Education and Humanities, Nanjing Sport Institute, Nanjing, China

**Keywords:** health outcomes, outdoor fitness equipment, physical activity, physical fitness, psychological health, systematic review

## Abstract

**Background:**

Outdoor fitness equipment (OFE) is an environmental infrastructure in public areas to facilitate structured physical activity. This systematic review aims to evaluate the effectiveness of OFE intervention on health outcomes.

**Methods:**

We searched five online databases (PubMed, Scopus, Web of Science, Embase, and The Cochrane Library) from inception to Nov 3, 2025. Randomized and non-randomized controlled studies that employed OFE interventions were included. We assessed methodological quality using the modified Downs and Black checklist. Primary outcomes included physical activity, physical fitness, psychological well-being, and other health-related results.

**Results:**

Fourteen studies underwent systematic review, of which eleventh provided sufficient data for meta-analyses, and 11 were rated as high quality. The OFE intervention notably increased physical activity. Significantly improvements were observed in physical fitness, including cardiorespiratory fitness (SMD = 0.53), lower limb muscle strength (SMD = 0.35), upper limb muscle strength (SMD = 0.25), and balance (SMD = 0.83). Improvements were also evident in psychological health, including mental well-being (SMD = 0.48), loneliness (SMD = 0.33), depression, stress symptoms, and self-efficacy. Furthermore, there were significant advancements in health-related issues such as quality of life (SMD = 1.06), fall risk (SMD = 1.01), hypertension, hypoglycemia, and blood lipid levels.

**Conclusion:**

The findings provide a comprehensive overview of the effectiveness of OFE intervention on health outcomes. The evidence indicates that OFE intervention effectively improves physical activity, physical fitness, psychological health, and health-related issues. High-quality RCTs are required to determine effective OFE intervention protocols and facilitate translation into practice.

**Systematic review registration:**

https://www.crd.york.ac.uk/PROSPERO/view/CRD42023449759, CRD42023449759.

## 1 Introduction

Physical inactivity is a worldwide issue with significant health and social risks (1, 2). It causes 6–10% of non-communicable disease-related deaths (2) and higher percentages of specific illnesses (e.g., 27% of diabetes) (3). The considerable incidence of physical inactivity significantly increased the government’s financial burden, and it was reported that physical inactivity caused a $67.5 billion economic loss in 2013 (1). Considering this dire situation, the “Global Action Plan on Physical Activity 2018–2030: More Active People for a Healthier World,” which was aimed at active societies, active environments, active people, and active systems, was launched by the World Health Organization (WHO) (4). It was suggested that infrastructure, policy, advocacy, and surveillance were crucial for realizing the plan’s objectives outlined above (5, 6). Among the suggestions, facilities for physical activity were studied as the most effective strategy to combat physical inactivity at the population level (7). Therefore, accelerating infrastructure is a reasonable and feasible solution to addressing this issue.

Outdoor fitness equipment (OFE) is an accessible environmental infrastructure constructed in open spaces, providing free opportunities for structured physical activity. It consists of simple, durable exercise machines or stations designed to provide organized physical activity (8–10). Historically, the origins of modern OFE can be attributed to the innovative ideas of Swiss architect Erwin Weckerman in the 1960s, whose visionary approach involved incorporating simple fitness equipment into walking paths, serving as a prototype for future OFE advancements. With the increasing awareness of fitness and the urgent need for space optimization, the variability of OFE is expanding. Some were designed for a specific population (e.g., the older adult) to meet the complex demands (11). Some were combined with other facilities (e.g., walkways) to increase the diversity and intensity of physical activity (12). Thus, various terminologies are used to describe OFE, including “open gyms” (13), “golden age gym” (14), and “fitness zones” (15). For consistency, the term “outdoor fitness equipment” and the abbreviation “OFE” will be utilized throughout the text.

Early examples of research into OFE aimed to investigate its promotion of physical activity, and some utilized observational methods. The findings indicate that exercise time on moderate-to-vigorous physical activity increased after installing OFE (9, 16). Nowadays, OFE has become prevalent globally, with installations in countries including Australia (17), the United States (16), Colombia (18), and China (8). Due to the popularity and diversity of OFE, the focus of OFE research engaged in more dimensions to evaluate the efficacy of qualitative studies, such as physical fitness, psychological well-being, and other health-related outcomes. For instance, Sales et al. employed interview methods to assess the impact of OFE intervention on individuals’ health outcomes (19). The research outcomes exhibited positive effects of the intervention on the participant’s muscle strength, balance, flexibility, confidence, and social interaction. Given its wide range of health benefits, OFE is recognized by society as a health intervention.

With the growing number of studies investigating the effects of OFE, several reviews have been conducted to summarize its impacts. Initially, most studies utilized qualitative research methods, leading to an integrative synthesis in previous reviews. Lee et al. synthesized evidence from quantitative, qualitative, and mixed methods studies and found that pursuing health and socialization opportunities was the primary motivation for using OFE (20). Meanwhile, Jansson et al. noted that while OFE may enhance physical activity, fitness, and other health-related outcomes, there was a lack of experimental studies supporting these findings (21). As more randomized controlled trials (RCTs) were conducted, meta-analyses emerged to assess the effects of OFE quantitatively. Ng et al. performed a mixed-methods meta-analysis to examine the impact of OFE on health outcomes in older adults (22). The results indicated that OFE did not improve physical performance, and whether OFE effectively increased physical activity remained inconclusive. While some evidence suggests a positive influence on specific health outcomes, several unanswered questions remain.

Researchers have not treated OFE in much detail, which might be the reason for the inconclusiveness. Firstly, previous reviews included various types of studies (prospective, cross-sectional, qualitative, pre-post, and RCTs) and lacked quantitative data, making it challenging to draw clear conclusions due to the complexity of OFE. Secondly, the heterogeneity of study types has led most existing reviews to favor systematic reviews and integrative synthesis over meta-analyses, which typically provide stronger evidence. Thirdly, the limited number of RCTs has hindered comprehensive meta-analyses, as only two were included in the existing meta-analysis. Given the limitations mentioned above, and to compensate for the lack of RCTs in the meta-analysis conducted by Ng, this research provided a comprehensive screening and analysis of OFE-related quantitative studies. It was significant in discovering the effectiveness of OFE on health outcomes.

Therefore, the primary objective of this systematic review was to assess the impact of OFE intervention on various aspects of health. These include physical activity (i.e., changes in physical activity levels before and after OFE intervention), physical fitness (i.e., indicators of strength, balance, and cardiorespiratory fitness measured through tests), psychological health (i.e., indicators of depression, loneliness, and anxiety measured using scales), health-related issues (i.e., any health outcome such as diabetes, hypertension, falls risk, and quality of life). We aim to address these critical questions through a rigorous and structured analysis: Do OFE intervention effectively promote physical activity? Can they lead to significant improvements in health and well-being?

## 2 Materials and methods

This systematic review followed the Preferred Reporting Items for Systematic Reviews and Meta-Analyses (PRISMA) guidelines (23) and was registered in the International Prospective Register of Systematic Reviews (Prospero registration: CRD42023449759).

### 2.1 Search strategy

We searched five online databases (Web of Science, Scopus, PubMed, Embase, and the Cochrane Library) from inception to May 24, 2024, with an updated search conducted on November 3, 2025. In addition, a manual search of the reference lists of included studies, relevant systematic reviews, and meta-analyses was performed to identify additional eligible studies. Search terms included a combination of keywords associated with OFE (e.g., outdoor gym, fitness zone, open gym, stretch station, public fitness facility, older adult fitness corner, senior exercise park, active park) and outcomes (e.g., effect, impact, assess, measure). The entire search strategy is available in Supplementary file 1.

### 2.2 Eligibility criteria

Inclusion criteria:

Participants: No restrictions.

Interventions: Interventions delivered through OFE (i.e., OFE installed in outdoor community parks or Seniors Exercise Park) that aim to improve health outcomes (e.g., physical fitness and/or psychological well-being and/or health-related outcomes).

Comparison: The comparator groups were strictly defined as ‘treatment as usual’, ‘wait-listed’, or non-active controls. Participants in these groups were instructed to maintain their habitual lifestyle and did not engage in any structured physical activity or exercise. Importantly, these groups did not receive any generic physical activity advice or educational materials during the intervention period.

Outcome: Outcomes included physical activity levels with objective measures (e.g., accelerometers, step counters) or subjective assessments (e.g., the Physical Activity Scale for the Elderly Questionnaire), physical fitness (e.g., balance, strength, mobility, and endurance), psychological health (e.g., depression, loneliness, and anxiety), and health-related issues (e.g., hypertension, hypoglycemia, falls risk, and quality of life). Studies were excluded if they did not report at least one of these outcome measures.

Study Design: RCTs and non-randomized controlled trials (non-RCTs) were included.

Exclusion criteria: Studies will be excluded if they meet the following criteria: unable to extract study data; a review, study protocol, case study, or conference paper; the OFE intervention group included other confounding factors such as medications; The comparison group involved some form of physical activity or exercise; non-English language studies.

### 2.3 Study selection and data extraction

We utilized Endnote X9 software to remove duplicates from the retrieved literature. After removing duplicates, two reviewers independently screened each article (ZY. T. and YQ. J.) for the title and abstract. Full-text versions of the remaining articles were assessed for eligibility after removing irrelevant studies.

Two reviewers (ZY. T. and YQ. J.) independently extracted data using a predesigned extraction form. Data extraction included: Basic literature details (first author name, publication year, country, and study design); participant characteristics (mean age, sex, sample population, and sample size); Characteristics of the OFE (terminology, locations, No. of OFE, and type of exercise); intervention components (type, session time, frequency, duration, outcome measured); significant findings (physical activity and/or physical fitness and/or psychological health and/or health-related issues). Disagreements were resolved by consensus or by a third reviewer (YS. Z.) at each stage of the screening process.

### 2.4 Risk of bias assessment

Two reviewers (ZY. T. and HB. X.) independently assessed the methodological quality of the included studies based on a modified Downs and Black checklist. The checklist demonstrated high test–retest and good inter-rater reliability for assessing the methodological quality of RCTs and non-RCTs (24, 25). Consistent with previous systematic reviews, the modified checklist consists of 27 items divided into five subscales: reporting (10 items), external validity (3 items), internal validity-bias (7 items), internal validity confounding (6 items), and statistical power (1 item) (26, 27). The checklist scores range from 0 to 28. The methodological quality of a study was classified into four levels: poor quality with a score ≤ 14; fair quality if it scored 15–19; good quality if it scored 20–25; and excellent quality if it scored 26–28 (28). Discrepancies between reviewers about the methodological quality of the included studies were resolved by a third reviewer (YS. Z.).

### 2.5 Data analysis

Among the inclusion, non-RCTs compared baseline and post-intervention outcomes, and RCTs compared the difference in value of pre- and post-intervention results between intervention and control groups. Data analysis was conducted using Stata 14.0 software (Stata, Texas, United States). The included data were continuous variables and effect sizes (ESs) were calculated using the standardized mean difference (SMD) with 95% confidence intervals (CI). In cases with insufficient data for meta-analyses, the magnitude of ESs was calculated using Hedges’ g (29, 30). Effect thresholds were defined as follows: 0.2, 0.5, and 0.8, representing small, medium, and large effects, respectively (31). Heterogeneity was assessed using the *p*-value (with a threshold of 0.1) and the I2 statistic (with points of 25, 50, and 75% representing small, medium, and large ratios of inter-study heterogeneity) (32). The fixed-effect model was applied if no statistical heterogeneity was found across studies (I^2^ ≤ 50%, *p* > 0.1). Otherwise, the random-effects model was utilized (33). Publication bias (e.g., Egger’s regression test) was not performed because fewer than 10 studies were included in each analysis. Sensitivity analysis was performed by excluding individual studies one by one using Stata 14.0 software. In addition, for a study that included two interventions that were distinguished by a and b.

### 2.6 Evidence certainty assessment

The quality of evidence for each outcome was assessed by two independent researchers (HB. X. and YQ. J.) using the Grading Recommendations to Assess Development and Evaluation System (GRADE), an internationally recognized standard for evaluating evidence quality and classifying recommendation strength (34). The quality of evidence is categorized into four levels: I (high), II (moderate), III (low), and IV (very low). Several factors can decrease evidence quality, including the risk of bias, imprecision, inconsistency, indirectness, and publication bias. Conversely, evidence quality can be increased by factors such as effect size, dose–response gradient, and control of confounding variables. For more information, please refer to Supplementary file 2.

## 3 Results

### 3.1 Literature search results

**Figure 1:**
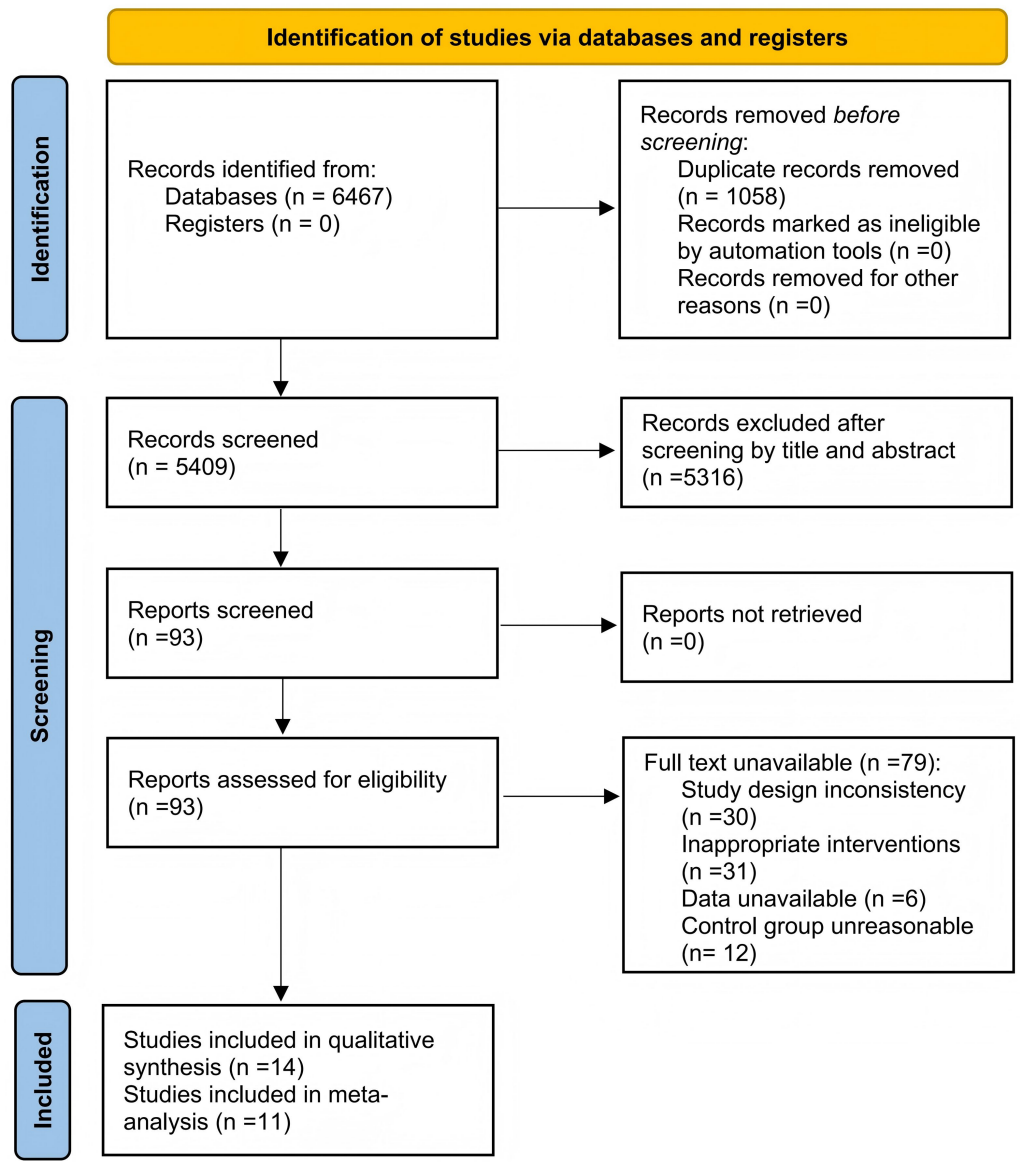
PRISMA flow diagram of the selection.

A total of 6,467 articles were identified from five databases (Web of Science: 1,323; Scopus: 3,953; PubMed: 446; Embase: 696; Cochrane Library: 49) in the initial search. After 1,058 duplicate articles were removed, 5,409 were screened by titles and abstracts. Subsequently, 93 articles were retrieved through full-text screening, of which 14 were eligible for inclusion. Finally, the systematic review included 14 articles, while the meta-analysis included 11 (35–45). Among the studies included in the meta-analysis, three non-RCTs conducted a single-arm meta-analysis (46–48). The screening process of the study is shown in Figure 1.

### 3.2 Description of included studies

All included studies are quantitative study with eight RCTs and six non-RCTs, the characteristics of the included literature is shown in Table 1. Four studies were conducted in Australia (40, 43–45), three in China (37, 41, 47), and the remains were from Brazil (35, 36), Sweden (46), Korea (38), Spain (39, 42), and the United States (48). Two-thirds of the studies were conducted within the past 5 years. A total of 828 participants were included, comprising 615 in the RCTs and 213 in the non-RCTs. The sample size ranged from 6 to 245, with participants aged 40 to 83. Specially, 32.6% of the participants were male.

**Table 1:** Characteristics of the included literature.

First author, year, Country	Study design	Participant characteristics		No. of OFE and type of exercise	Intervention using OFE	Supervision	Adherence	Intensity	Outcome measured	Main findings
		Mean age; sex-M%;	Sample size (IG/CG)							
Baruki et al. (36); Brazil ^b^	RCT	40–70; M-3.4%	10/10/9	7 Aerobic and resistance	STG and CTG: 16 × 3 × 50–60 min/ week	Researcher	NR (Dropout if >3 absences)	40–70% HRR/RPE 11–13	II: 6-Minute Walk Test; Sit-to-Stand; elbow flexion; Sit-and-Reach IV: SF-36; SBP; DBP	BMI ^STG^ ^a^ SR ^STG^ ^a^ DBP ^STG^ ^a^ SR ^CTG^ ^c^ mental control ^CTG^ ^c^
Barbosa et al. (35); Brazil ^b^	RCT	≥60; M-100%	20/20/20	4 Aerobic and resistance	SUTG and UTG:12 × 3 × 30 min/week	STG: experienced trainers UT: Unsupervised C: Unsupervised	SUTG: 100% UTG: 75%	SUTG: Metronome (30 reps/min) UT: Self-selected	I: IPAQ - SF II: W10 m; RSP; RVDP; SRCW; Body mass; BMI III: WHOQOL	W10 m ^SUTG^ ^a^ RSP ^SUTG^ ^a^ RVDP ^SUTG^ ^a^ SRCW ^SUTG^ ^a^
Chow et al. (37); China	non-RCT	66 ± 4.3; M-37.9%	29	7 Aerobic, resistance, balance, and flexibility	aerobic Training: 0–12 weeks, 5 × 40 min/week Resistance, Balance, and Flexibility Training: 12–24 weeks, 2 × 40 min/week	Coach	NR	Moderate (measures NR)	II: 2 min step test; 30s Chair stand test; handgrip strength test; Rom berg test, Single-leg stance test, 8-Foot Up- and-Go; ROM, Back Scratch	2 min step ^a^ handgrip strength ^a^ 30s Chair stand ^a^ back-scratch test ^c^ Single-leg stance test ^a^ 8-Foot Up- and-Go ^c^
Johnson et al. (46); Sweden	non-RCT	41.2 ± 6.5; M-66.7%	6	6 Aerobic and resistance	6 × 2 × 2/3/4 circuit/week	instructor	100%	Self-selected	I: Steps II: Bruce Treadmill Test; 1RM strength test III: GHQ-12; BREQ-2	steps ^a^ time to exhaustion ^a^ Upper limb muscle strength ^a^ stress symptoms ^a^
Kim et al. (38); Korea ^b^	RCT	73.2; M-8.57%	10/12/13	5 Aerobic and resistance	RTG: 6 × 3 × 50 min/week, CTG: 6 × 3 × 70 min/week,	Researcher	RTG: 75% CTG: 81%	moderate-vigorous (RPE 6–8)	II: 30 s chair stand, 30 s arm curl, 244 cm up and go, one-leg stand, and 2 min step; push-ups; six-min walk tests IV: fasting glucose; fasting insulin; Insulin resistance; IL-6 levels	push-up ^RTG^ ^a^ 6-min walk ^RTG^ ^a^ push-up ^CTG2^ ^a^ 6-min walk ^CTG^ ^a^ step test ^CTG^ ^a^
Lee et al. (47); China	non-RCT	75.63; M-17.4%	46	NR Aerobic, resistance, and balance	NR	Researcher	90.87%	Moderate (measures NR)	I: the fulfillment of the WHO PA recommendations; RAPA II: 30 s chair stand test; Sharpened Roberg test III: SEE-C Scale; The 23-item Chinese Barriers to Exercise scale IV: hypertension	balance ^a^ self-efficacy for exercise ^a^ exercise barriers ^a^ hypertension ^a^
Leiros-Rodríguez et al. (39); Spain ^b^	RCT	68.5; M-0%	14/14	12 Static balance and dynamic balance	12 × 2 × 50 min/week, balance training based on 12 OFE	physical therapist	66.7%	intermittent (60s Work / 60s Rest)	II: BBS, TUG III: SF-12	Berg Balance Scale ^a^ timed up-and-go test ^a^ SF-12 ^a^
Liu et al. (41); China ^b^	RCT	70; M-28.6%	21/21	3 Aerobic, resistance, and flexibility	12 × 3 × 40-45 min, structure training	three certified athletic trainers	100%	Aerobic: Metronome (120 bpm)	I: 2-min step test; back scratch; chair sit and reach tests; arm curl test; sit to stand test; timed up and go test	no significant improvement in all parameters ^c^
Levinger et al. (40); Austria ^b^	non-RCT	72.8; M-18.7%	80	22 Resistance, balance, flexibility, coordination, functional training	18 × 2 × 45–75 min/week, structure training	1–12 week: Accredited Exercise Physiologist or Physiotherapist 13–18 week: Unsupervised	86%	Supervised Dynamic Adjustment	I: CHAMPS II: 30-s sit-to-stand test; step test; two-minute walk test; 4 m walk test III: WHO-5 GDS-15; PACES, LSNS6, UCLA 3; SEE IV: EQ-5D-5L; Short FES-I; FROP-Com	physical activity (caloric expenditure, frequency per week, and total time) Sit to stand ^a^ Two-minute walk ^a^ step test ^a^ mental well-being ^a^ depressive symptoms ^a^ loneliness ^a^ Social isolation ^c^ self-efficacy for exercise ^c^ Enjoyment ^a^ quality of life ^a^ fear of falls ^a^ falls risk ^a^
Marcos-Pardo et al. (42); Spain ^b^	RCT	50-77; M-35.9%	64/64	8; Resistance, aerobic and flexibility	8 × 2 × 60 min/week	Two sports scientists	92.75%	Moderate (measures NR)	II: Body mass; MVIC elbow flexion test; MVIC leg extension test; 4 m gait walk; TUG IV: IF-36	Lean mass index ^a^ MVIC right leg ^a^ MVIC left leg ^a^ MVIC arms ^a^ TUG ^a^ 4 m gait walk ^a^
Ng et al. (43); Austria ^b^	non-RCT	77.8 ± 6.0; M-59%	46	22 Resistance, balance, flexibility, coordination, functional training	24 × 2 × 60 min/week, structure training	1–18 week: physiotherapist (1:8) 19–24 week: Unsupervised	1–18 week: 90.9% 19–24 week: 26.3%	Supervised Dynamic Adjustment	I: PASE II: CBMS-Home; The Five Times Sit to Stand Test; Four square step test III: WHO-5; UCLA; SEE IV: The Modified Falls Efficacy Scale; EQ-5D-5L	physical activity ^a^ balance and mobility ^a^ lower body strength ^a^ fast walking speed ^a^ psychosocial well-being ^a^ quality of life ^a^
Nguyen et al. (48); USA	non-RCT	35.0 ± 9.0; M-0%	6	6 Aerobic, resistance	6 × 3 × 60 min/week	researcher	100%	Aerobic: Moderate (measures NR) Resistance: Metronome (30 reps/min)	II: one-mile walk; push-up test, squat test, and curl-up test	muscular endurance repetitions ^a^ VO2max ^c^
Plotnikoff et al. (44); Austria ^b^	Cluster RCT	53.4 ± 13.9; M-28%	123/122	8 Resistance and aerobic	12 × 2 × 45-55 min/week	Unsupervised	92%	Multi-level (Self-selected)	I: accelerometers; Godin Leisure-Time questionnaire; the Global Physical Activity Questionnaire II: 90-degree push-up test; 60-s sit-to-stand test; 3-min YMCA step test III: depression, anxiety, and stress scale; physical activity self-efficacy scale	Self-reported total MET ^c^ upper and lower body muscular fitness ^c^ enjoyment, self-efficacy, and implementation intention for resistance training ^a^
Sales et al. (45); Austria ^b^	RCT	71.4; M-29.0%	21/27	16 Balance, coordination, strength, mobility, range of motion	16 × 2 × 45–75 min/week, structure training	accredited exercise physiologist	79.6%	light to moderate	II: BOOMER; the single leg stance test, hand grip; 2-min walk test; 30-s sit-to-stand test; gait speed IV: SF-12; FES-I	single-leg stance ^a^ knee strength ^a^ 2-min walk ^a^ timed sit-to-stand test ^a^

BREQ-2, Behavioral Regulation in Exercise Questionnaire-2; BBS, the Berg Balance Scale; BOOMER, The Balance Outcome Measure for Elder Rehabilitation; CHAMPS, The Community Healthy Activities Model Program for Seniors; CTG, physical training in circuit group; CBMS-Home, The Community Balance and Mobility Scale-Home; DBP, the Diastolic Blood Pressure; FES-I, The Short Falls Efficacy Scale International questionnaire; FROP-Com, The Falls Risk for Older People in the Community; GHQ-12, The General Health Questionnaire–12; GDS-15, Geriatric Depression Scale; HR, Heart Rate; HRR, Heart Rate Reserve; IL-6, Interleukin-6; LSNS6, 6-item Lubben Social Network Scale; NR, no report; PACES, Physical Activity Enjoyment Scale; PASE, The Physical Activity Scale for the older adults Questionnaire; RPE: Rating of Perceived Exertion Scale; RSP, rising from a sitting position; RVDP, rising from the prone position; ROM, range of motion; RAPA, Rapid Assessment of Physical Activity; STG, physical training in sets group; SEE-C Scale, The 9-item Chinese self-efficacy for exercise scale; SF-12, Short Form 12 Health Survey; SS, Sit to Stand; SR, the Sit and Reach; SBP, the Systolic Blood Pressure; SF-36, 36-Item Short Form Health Survey; SUTG, Supervised training group; SRCW, sitting and rising from the chair and move around the house; TUG, the Timed Up and Go test; UTG, Unsupervised training group; WHO-5, the five-item World Health Organization Well-being questionnaire; W10 m, walking 10 m.

a Significant statistical improvement.

b Studies included in the meta-analysis.

c No statistically significant change; I, physical activity; II, physical fitness; III, psychological health; IV, health-related issues.

Most OFE investigated were in public parks, and a few were in community open spaces. Given the significant variation in naming conventions across the literature, a detailed glossary listing the specific terminology (e.g., ‘fitness zones’, ‘seniors exercise parks’) and locations for each included study is provided in Supplementary file 3. Existing OFE can be categorized into several types of equipment that target various aspects of physical fitness. To provide a clearer overview of the interventions, a detailed classification of the specific equipment used in each study, including their targeted functions, adjustability, and progression features, is presented in Supplementary file 4. Cardio equipment, such as air walkers and ski machines, aim to improve cardiorespiratory fitness. Resistance equipment, such as rowing machines and Bonny riders, focuses on increasing muscular strength. Flexibility equipment, including arm stretches and shoulder wheels, promotes flexibility. Balancing equipment, such as waist twists, jumping boxes, and balance beams, seeks to enhance balance. Lastly, core equipment, such as push-up and pull-up bars, work on core skills. It is important to note that each type of equipment serves a specific purpose in overall health. OFE mentioned above has several limitations. One major limitation is the inability to adjust the equipment to fit the individual user’s needs, body shape, or size. Another limitation is the inability to modify the resistance level, which affects the ability to alter the intensity of the workout.

The interventions in this study targeted specific functions (e.g., cardiorespiratory training, balance training, aerobic training) or comprehensive functions (i.e., containing a variety of physical fitness). The frequency of interventions ranged from 2 to 3 times/week, with sessions lasting from 45 to 75 min and duration ranging from 6 to 24 weeks. Certain studies examined intervention intensity, measuring heart rate or ratings of perceived exertion (RPE) scales.

The efficacy of OFE interventions is assessed in various ways. Physical activity is generally measured with scales such as the Physical Activity Scale for the elderly questionnaire and the Physical Activity Rapid Assessment Scale. Physical fitness is assessed with functional tests such as grip strength, 30-s arm flexion, 30-s sit-to-stand, 2-min walking, one-legged standing, and Romberg test. Psychological health is evaluated with scales such as the General Mental Health Questionnaire, the Loneliness Scale, the Geriatric Depression Assessment Scale, and the Exercise Self-Efficacy Scale. Health-related issues are assessed with scales such as the Falls Risk for Older People in the community and the 36-Item Short Form Health Survey.

### 3.3 Assessment of study quality

The methodological quality of the included articles was assessed by the Downs and Black checklist, as presented in Table 2 and additional information in Supplementary file 5. Scores range from 19 to 24, with most articles rated as good (11/14, 78.6%) and three as fair (3/14, 21.4%). For the subscales, most of the articles met the reporting requirements (9/11 points), while external validity (1/3 points), bias (5.6/7 points), and confounding (4.9/6 points) were more or less problematic. Twelve (12/14, 85%) articles reported power analyses. The primary issues identified were the absence of randomization and blinding procedure information.

**Table 2:** Quality of included articles.

Study	Reporting	External validity	Internal validity—bias	Internal validity—confounding	Power	Total	Quality rating^a^
	11 points	3 points	7 points	6 points	1 point		
Baruki et al. (36)	9	1	7	6	1	24	Good
Barbosa et al. (35)	9	1	6	6	1	23	Good
Chow et al. (37)	9	1	5	4	1	20	Good
Johnson et al. (46)	9	1	5	4	0	19	Fair
Kim et al. (38)	9	1	6	5	1	22	Good
Lee and Ho (47)	8	1	5	4	1	19	Fair
Leiros-Rodríguez and García-Soidan (39)	9	1	5	5	1	21	Good
Levinger et al. (40)	9	1	5	4	1	20	Good
Liu et al. (41)	9	1	7	6	1	24	Good
Ng et al. (43)	9	1	5	4	1	20	Good
Nguyen and Raney (48)	9	1	5	4	0	19	Fair
Plotnikoff et al. (44)	9	1	5	5	1	21	Good
Marcos-Pardo et al. (42)	9	1	6	6	1	23	Good
Sales et al. (45)	9	1	7	6	1	24	Good

a Quality rating: Excellent (26–28), good (20–25), fair (15–19), and poor (≤14).

### 3.4 Health outcomes

#### 3.4.1 Physical activity

A total of five studies assessed the impact of OFE interventions on physical activity. Regarding outcomes derived from self-reported measures, three studies consistently demonstrated positive effects. Lee et al. observed a significant increase in the proportion of participants meeting WHO physical activity recommendations following the intervention (47). Similarly, Levinger et al. reported significant improvements in caloric expenditure, exercise frequency, and total exercise time using the CHAMPS questionnaire (40). Ng et al. also found a significant increase in physical activity levels assessed by the PASE questionnaire after an 18-week intervention, which was sustained at the 24-week follow-up (43).

By contrast, evidence from device-based measures employed in two studies yielded mixed findings. Johnson et al. utilized pedometers and identified a significant increase in daily step counts following a 6-week outdoor gym circuit program (46). However, Plotnikoff et al., employing accelerometers in a cluster RCT, did not observe significant changes in moderate-to-vigorous physical activity levels between the intervention and control groups (44). This discrepancy between measurement methods suggests that self-reported assessments may potentially overestimate the intervention’s impact on overall activity levels compared to objective monitoring.

#### 3.4.2 Physical fitness

##### 3.4.2.1 Cardiorespiratory fitness

**Figure 2:**
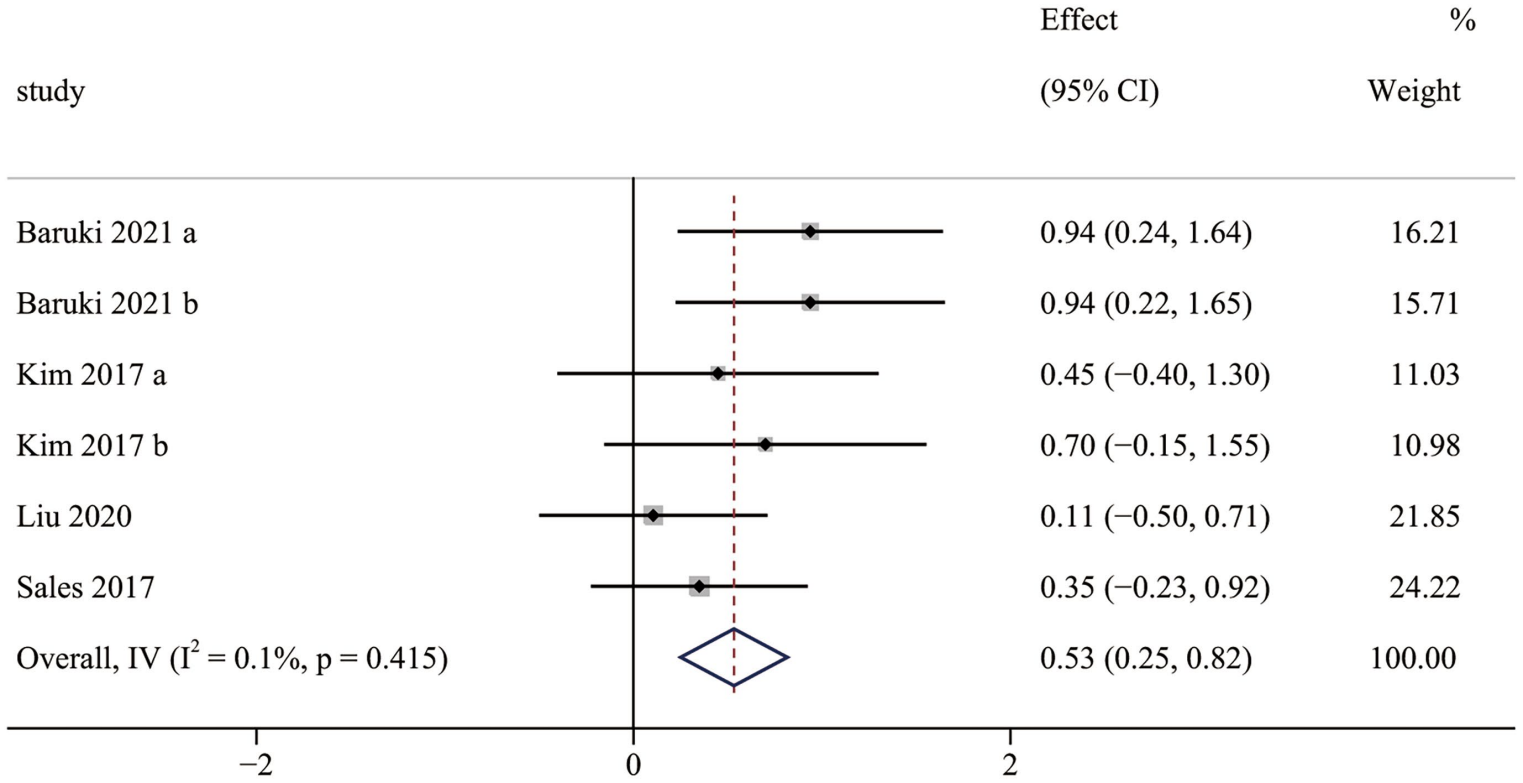
The effect of OFE intervention on cardiorespiratory fitness.

The cardiorespiratory fitness results contained four RCTs (36, 38, 41, 45), including six pairwise comparisons. Meta-analysis results indicated that OFE intervention was effective in improving cardiorespiratory fitness compared to controls (SMD = 0.53; 95% CI (0.25 to 0.82); *p* < 0.001; Figure 2). No significant heterogeneity was observed in the meta-analysis results (I^2^ = 0.1%, *p* = 0.415). In addition, three non-RCTs (37, 40, 46) reported that OFE intervention significantly improved cardiorespiratory fitness.

##### 3.4.2.2 Lower limb muscle strength

**Figure 3:**
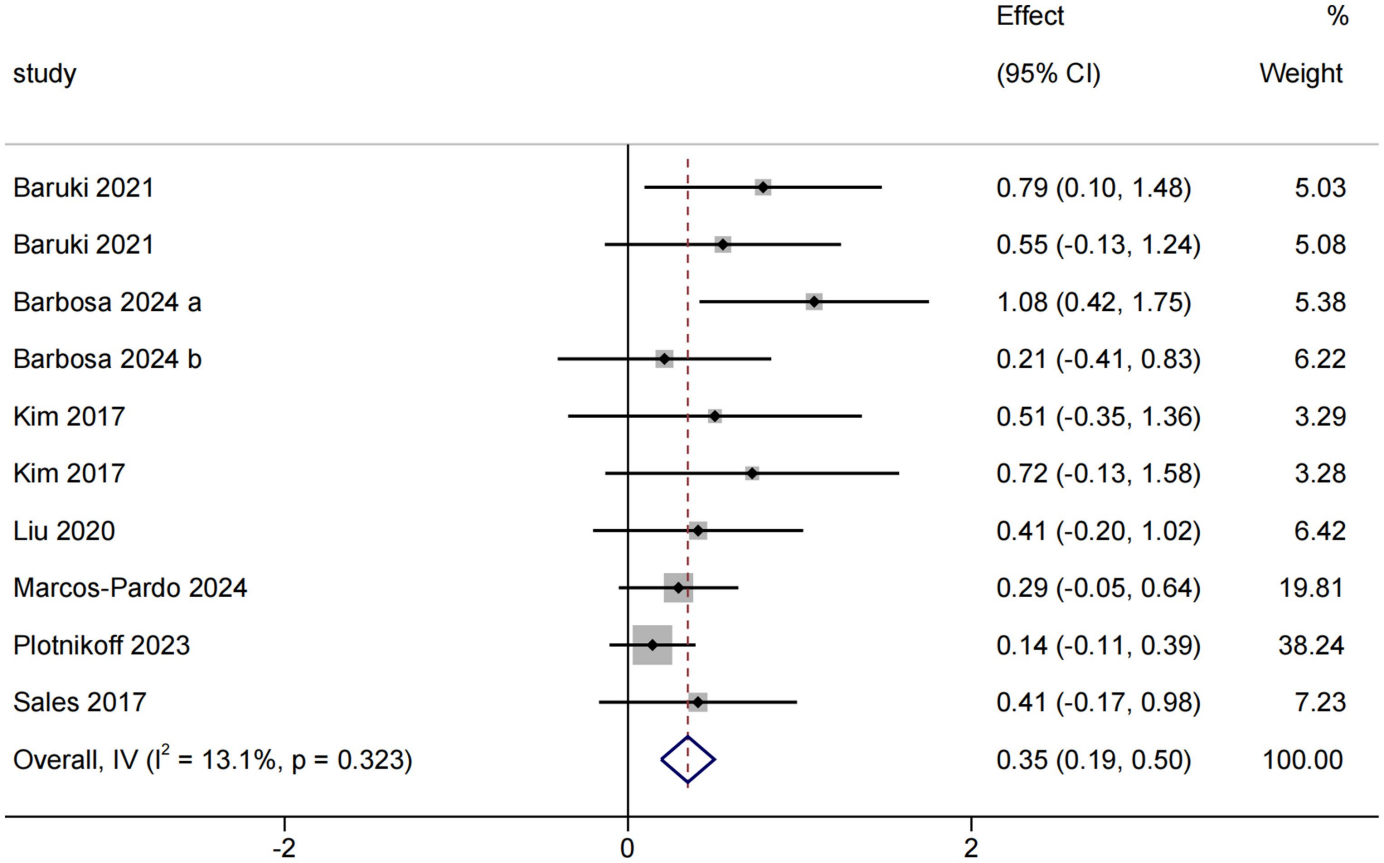
The effect of OFE intervention on lower limb muscle strength.

The lower limb muscle strength results contained seven RCTs (35, 36, 38, 41, 42, 44, 45), including 10 pairwise comparisons. Meta-analysis results indicated that OFE intervention was effective in improving lower limb muscle strength compared to controls (SMD = 0.35; 95% CI (0.19 to 0.50); *p* = 0.001; Figure 3). No heterogeneity was observed in the meta-analysis results (I^2^ = 13.1%, *p* = 0.323). In addition, five non-RCTs (37, 40, 43, 46, 48) reported that OFE interventions significantly improved lower limb muscle strength.

##### 3.4.2.3 Upper limb muscle strength

**Figure 4:**
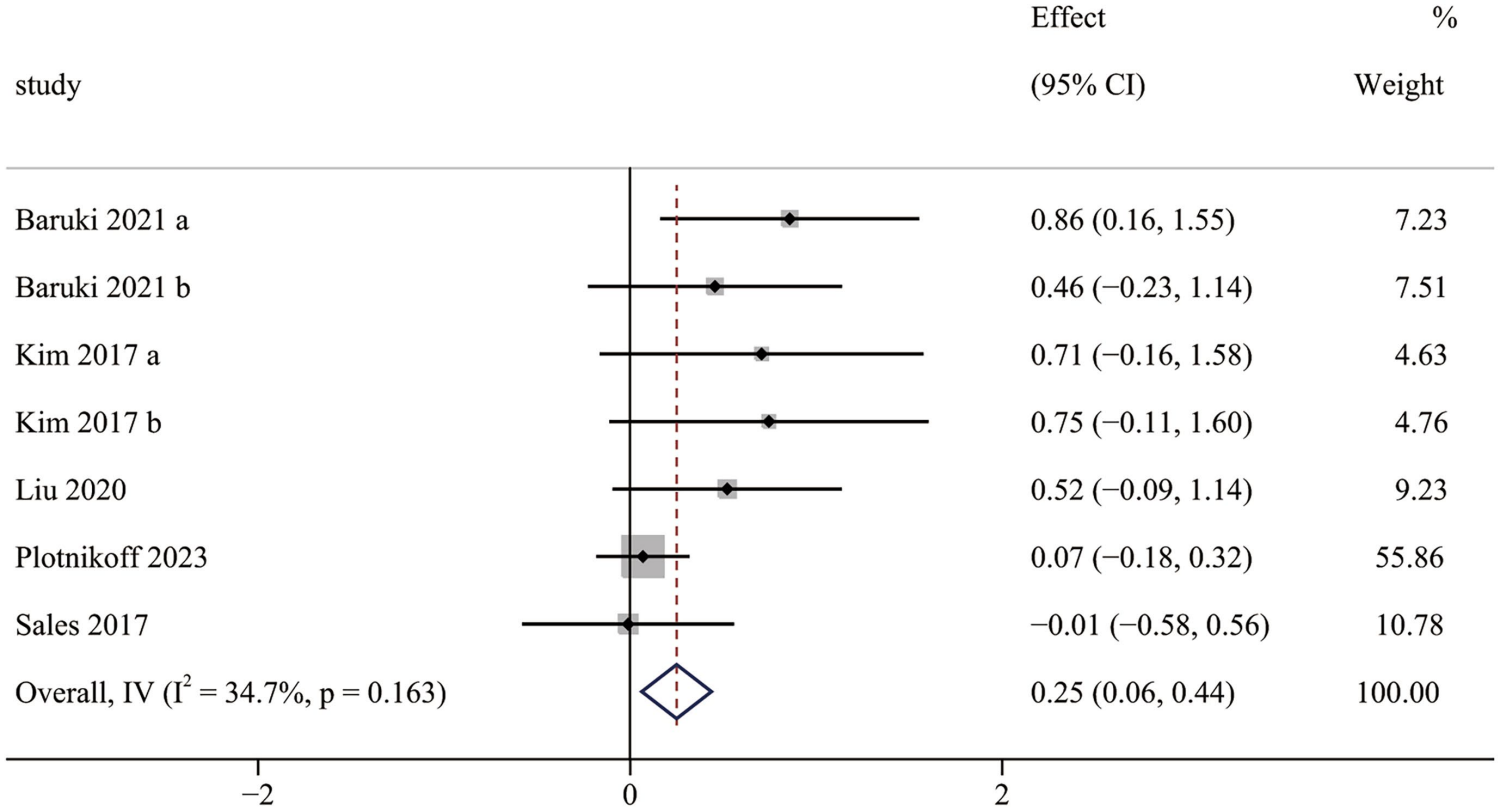
The effect of OFE intervention on upper limb muscle strength.

The upper limb muscle strength results contained five RCTs (36, 38, 41, 44, 45), including seven pairwise comparisons. Meta-analysis results indicated that OFE intervention was effective in improving upper limb muscle strength compared to controls (SMD = 0.25; 95% CI (0.06 to 0.44); *p* = 0.009; Figure 4). Small heterogeneity was observed in the meta-analysis results (I^2^ = 34.7%, *p* = 0.163). In addition, two non-RCTs (46, 48) reported that OFE intervention significantly improved upper limb muscle strength.

##### 3.4.2.4 Balance

**Figure 5:**
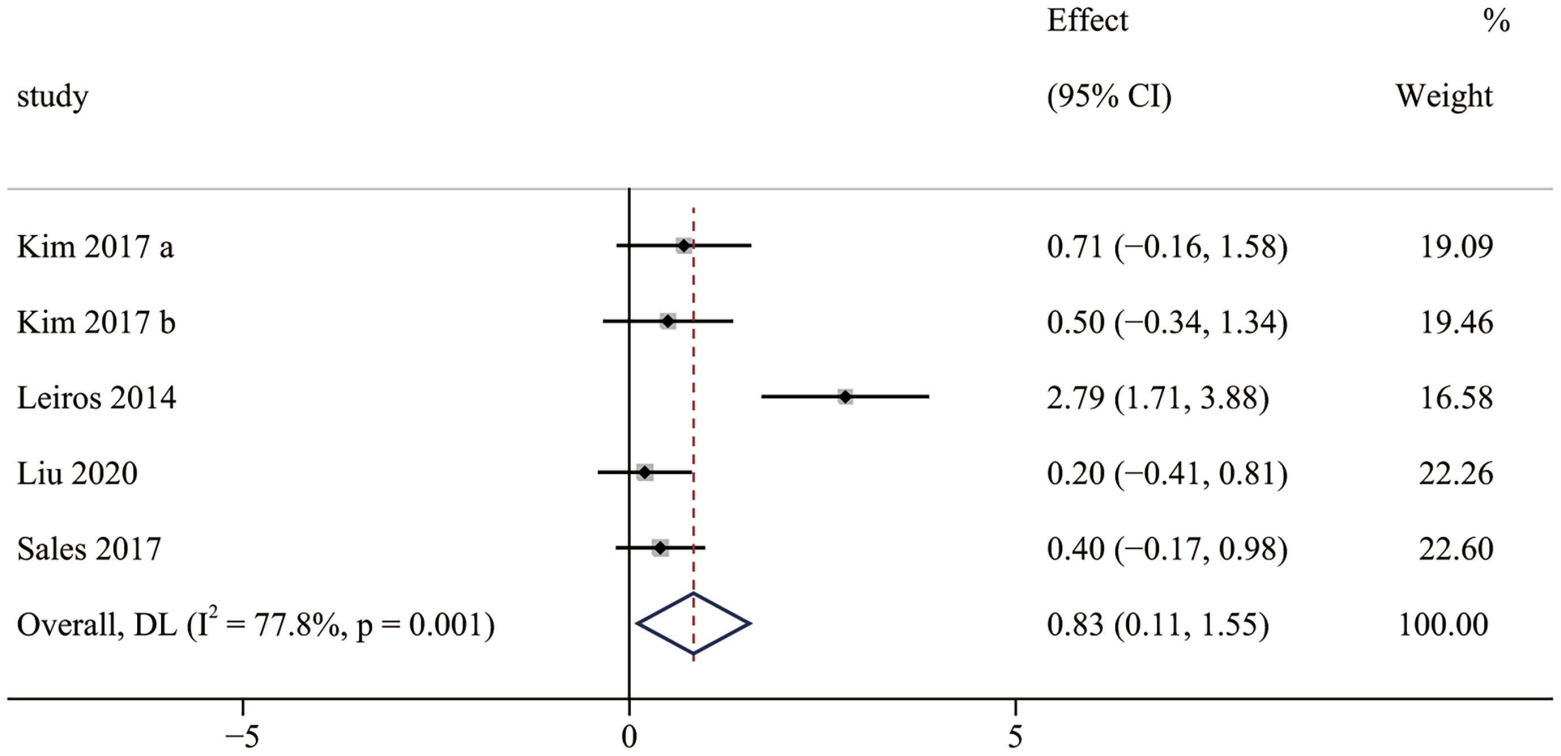
The effect of OFE intervention on balance.

The balance results contained four RCTs (38, 39, 41, 45), including five pairwise comparisons. Meta-analysis results indicated that OFE intervention improved balance more effectively than controls (SMD = 0.83; 95% CI (0.11 to 1.55); *p* = 0.024; Figure 5). Significant heterogeneity was observed in meta-analysis results (I^2^ = 77.8%, *p* = 0.001). Subgroup analyses were conducted to identify heterogeneity sources (Supplementary Figure 1). The subgroup analysis indicated that the choice of measurement tools significantly influenced the effect of OFE intervention. The different measurement tools might be the source of heterogeneity. In addition, three non-RCTs (37, 43, 47) demonstrated that OFE interventions significantly improved balance.

#### 3.4.3 Psychological health

##### 3.4.3.1 Mental well-being

**Figure 6:**
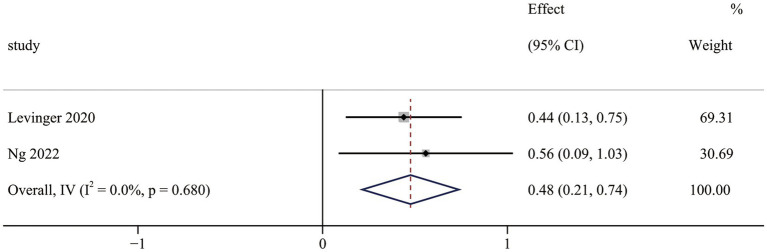
The effect of OFE intervention on mental well-being.

The mental well-being results contained two non-RCTs (40, 43). Meta-analysis results indicated that OFE intervention was effective in improving mental well-being compared to controls (SMD = 0.48; 95% CI (0.21 to 0.74); *p* < 0.001; Figure 6). No heterogeneity was observed in the meta-analysis results (I^2^ = 0.0%, *p* = 0.680).

##### 3.4.3.2 Loneliness

**Figure 7:**
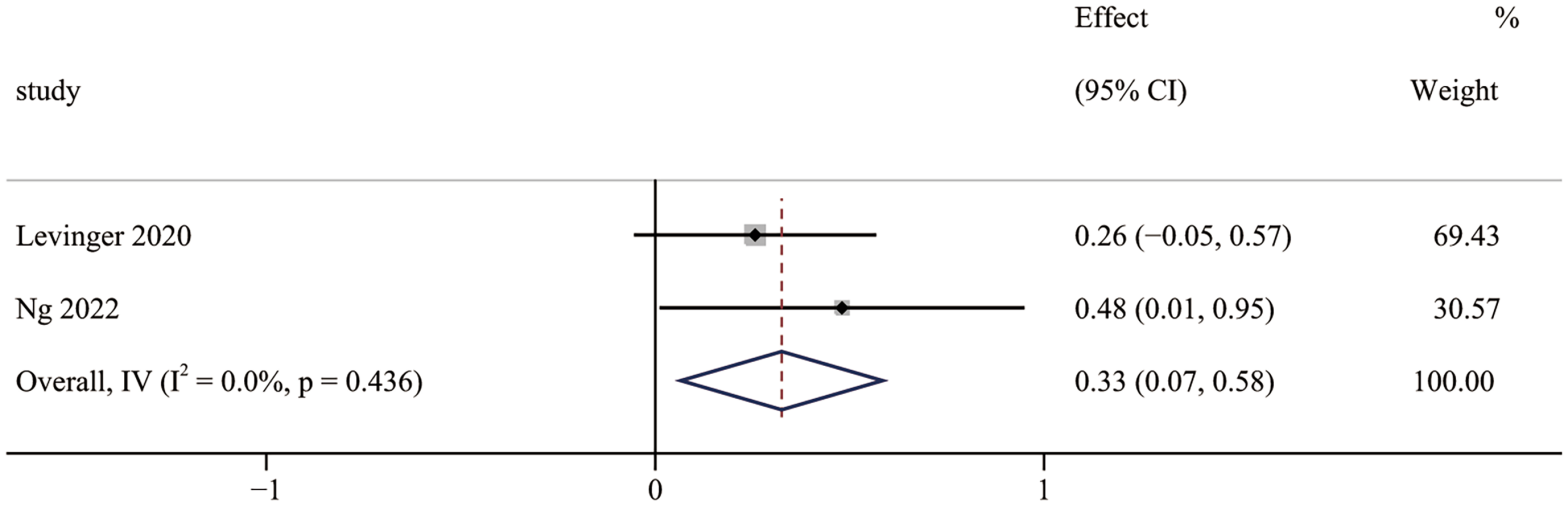
The effect of OFE intervention on loneliness.

The loneliness results contained two non-RCTs (40, 43). Meta-analysis results indicated that OFE intervention was effective in improving loneliness compared to controls (SMD = 0.33; 95% CI (0.07 to 0.58); *p* = 0.014; Figure 7). No heterogeneity was observed in the meta-analysis results (I^2^ = 0.0%, *p* = 0.436).

Four non-RCTs (40, 43, 44, 47) examined the effect of OFE intervention on individual self-efficacy. Of those, three studies reported that OFE interventions significantly improved self-efficacy in individuals. In contrast, one study reported that OFE interventions did not improve the individuals’ self-efficacy. In addition, three studies reported that OFE interventions significantly improved depression, stress symptoms, autonomous motivation, and enjoyment in individuals.

#### 3.4.4 Health-related issues

##### 3.4.4.1 Quality of life

**Figure 8:**
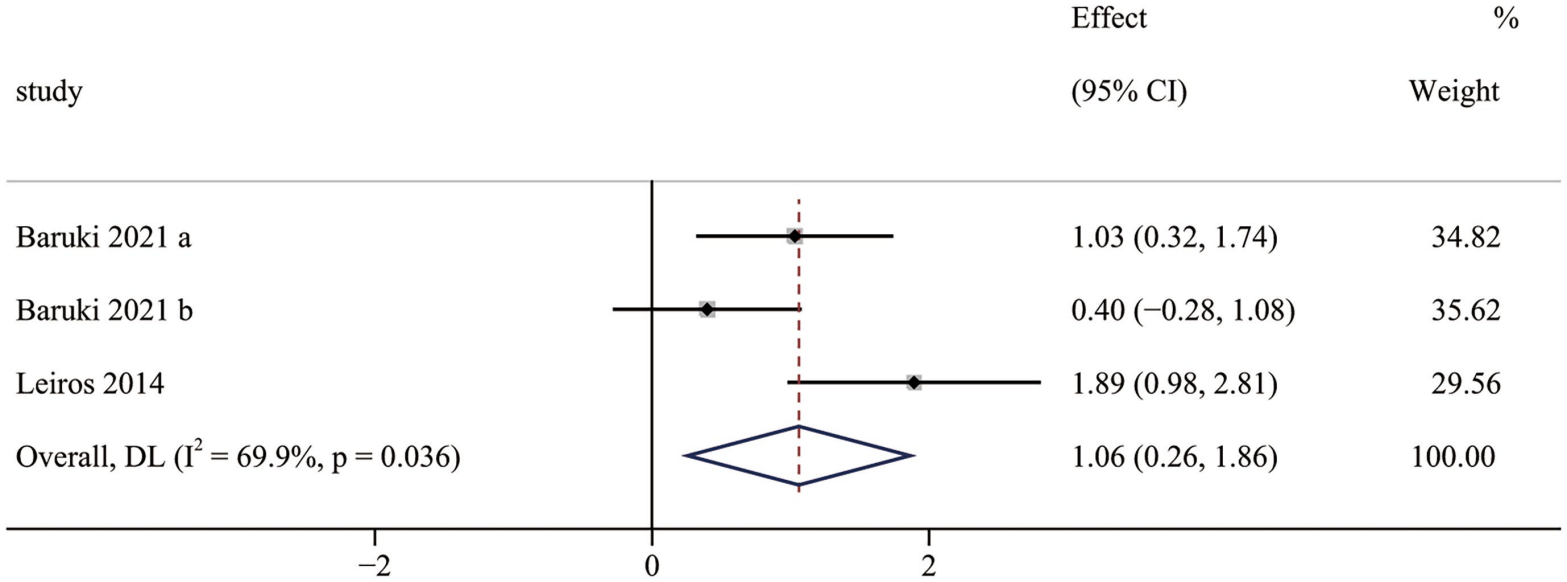
The effect of OFE intervention on quality of life.

Quality of life results contained two RCTs (36, 39), including three pairwise comparisons. Meta-analysis results indicated that OFE intervention improved quality of life more effectively than controls (SMD = 1.06; 95% CI (0.26 to 1.86); *p* = 0.010; Figure 8). Significant heterogeneity was observed in meta-analysis results (I^2^ = 69.9%, *p* = 0.036). Subgroup analyses were conducted to identify heterogeneity sources (Supplementary Figure 2). The subgroup analysis indicated that the choice of measurement tools significantly influenced the effect of OFE intervention. The different measurement tools might be the source of heterogeneity. In addition, two non-RCTs (40, 43) reported that OFE interventions significantly improved the quality of life.

##### 3.4.4.2 Falls risk

**Figure 9:**
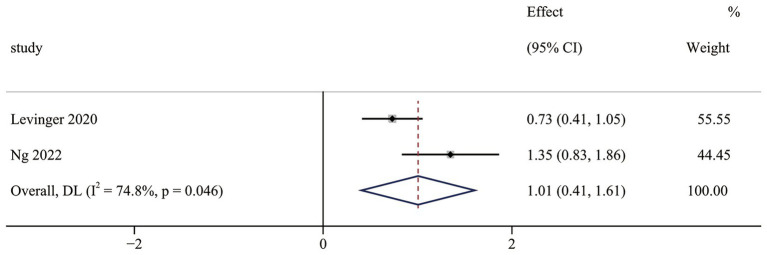
The effect of OFE intervention on falls risk.

The fear of falls results contained two non-RCTs (40, 43). Meta-analysis results indicated that OFE intervention was effective in improving fear of falls compared to controls (SMD = 1.01; 95% CI (0.41 to 1.61); *p* = 0.001; Figure 9). Significant heterogeneity was observed in meta-analysis results (I^2^ = 74.8%, *p* = 0.046). The results include only two trials that were not analyzed in subgroups, so the reasons for the heterogeneity remain unknown.

In addition, some studies have found that OFE interventions can positively affect hypertension, hypoglycemia, and blood lipid levels (38, 47).

### 3.5 GRADE quality evaluation

The GRADE evidence profile for RCT outcomes is presented in Supplementary file 6. Evidence quality ranges from moderate to high. Specifically, there is high evidence that OFE intervention significantly affects cardiorespiratory fitness and balance. In addition, there is moderate evidence that OFE intervention significantly affects lower limb muscle strength, upper limb muscle strength, and quality of life.

### 3.6 Sensitive analysis

To test the results’ stability and whether the heterogeneity is due to a single study. We used the one-to-one exclusion method for studies with high heterogeneity to analyze the combined effect sizes. Sensitivity analyses indicated that no single research influenced the combined effect estimate, affirming the robustness of our findings. For specific information, refer to Supplementary Figures 3–5.

## 4 Discussion

The current study aims to examine the effectiveness of OFE intervention on overall health. OFE interventions appear to positively affect physical activity, physical health, mental health, and health-related outcomes as assessed by validated measures.

### 4.1 Participant

It was found that users of OFE exhibit a distinctive age distribution. The subjects in previous research were exclusively middle-aged and older adults, ranging in age from 40 to 83, with a majority being older adult. OFE is typically uncomplicated in structure, adaptable in form, easy to use, and economical. It is likely due to such qualities that OFE has been widely embraced as a workout option for older adults. According to the World Health Organization, the global population of individuals aged 60 and above is expected to reach 2.1 billion by 2050 (49). Population aging and age imbalances will become significant global social issues. Given the rapid growth of aging populations, international experience indicates a transition in health management from a biomedical paradigm focused on disease treatment to a preventive medicine model emphasizing proactive intervention. As individuals age, vital bodily functions gradually deteriorate in motor, cognitive, and sensory capacities (50). As a result, a wide range of chronic diseases associated with aging arise. Therefore, establishing a health-focused exercise setting is critical to advancing healthy aging. This meta-analysis revealed that interventions based on OFE could considerably enhance physical performance, mental well-being, and quality of life. OFE was proved to be an efficient approach for cultivating healthy habits among older adults.

### 4.2 Physical activity

The benefits of OFE can be seen in increased physical activity time and considerably higher physical activity levels. Lee et al. employed a scale to evaluate the degree to which participants met WHO’s physical activity recommendation standards after OFE intervention (47). The results revealed a noteworthy rise in the number of participants who achieved the standards after the intervention. In addition, various studies have utilized the System for Observing Play and Recreation in Communities to assess the impact of installing OFE into parks on the population’s physical activity levels (16, 51). The findings demonstrated that combining OFE resulted in a rise in total METs compared to the baseline levels among park users. Moreover, there was a marked increase in moderate to vigorous intensity physical activity, coupled with a significant decrease in sedentary behavior (15). However, many physical activity measures presently used depend on self-reports or scales. These are likely to overestimate physical activity levels when contrasted to objectively measured levels. More significantly, OFE interventions also promote exercise habits and healthy lifestyles. The study revealed that 6 and 9 months after OFE intervention, participants’ physical activity remained significantly higher than baseline and exceeded the WHO recommendation of 150 min of moderate-intensity physical activity per week (47). Evidence indicates that individuals who participate in outdoor physical activity may be more compliant with regular exercise habits (52). In addition, it has been demonstrated that engaging in outdoor activities within green or natural environments can enhance the personal sense of vitality and active participation (53). Subsequently, OFE interventions can encourage individual physical activity and develop long-term healthy habits.

### 4.3 Physical fitness

The meta-analysis results demonstrate that OFE intervention has a small positive effect on upper limb muscle strength (SMD = 0.25) and lower limb muscle strength (SMD = 0.35), moderate positive effect on cardiorespiratory fitness (SMD = 0.53), and large positive effect on balance (SMD = 0.83). This finding contradicts previous meta-analyses, which concluded that OFE did not improve physical health (22). This may be due to the fact that the previous study limited the number of studies it analyzed, only calculating effect sizes for two. There were substantial differences between these two studies regarding session length, total duration, and exercise load. Moreover, the number of OFEs utilized and how outcome indicators were measured were inconsistent. Compared to prior research, the current meta-analysis expanded the number of studies analyzed and categorized them based on distinct aspects of physical health. The surveyed literature consists solely of high-quality randomized controlled trials, thereby strengthening the credibility and persuasiveness of the findings in this study. In addition, we also found that the OFE intervention was most effective in improving balance. OFE is often designed to promote functional training, which involves the coordinated use of different body parts (54). This integrated training method can be used to practice activities of daily living such as walking, standing, and turning. Therefore, balance and coordination skills may be challenged more often for people using OFE, contributing to improvements in balance skills. Cardiorespiratory fitness and muscular strength also improved but may have been influenced by the type and intensity of exercise, which was not as pronounced in the OFE intervention. More research may be needed to gain specific insights into the exact impact of OFE on these health outcomes.

### 4.4 Psychological health

This review indicates that OFE interventions have a beneficial impact on mental well-being. The OFE interventions positively affect mental health indicators, such as mental well-being, depression, stress symptoms, self-efficacy, and enjoyment. The meta-analysis results reveal that OFE interventions have a slight but positive effect on mental well-being (SMD = 0.48). The possible reason is that exercise modifies brain-derived neurotrophic factor (BDNF) levels, which can lead to improved mental health and brain plasticity (55). Furthermore, exercise forward endorphins release, which has been shown to alleviate symptoms of depression and anxiety (56, 57). Consistent with these findings, the present study revealed that the OFE intervention positively affected depression. Since exercising in a green or natural environment was proven to improve negative emotions in numerous studies, such as tension, anger, and depression (58), the placement of OFE in such settings may offer added value (59). As a result, stress symptoms of the OFE intervention are significantly reduced while both self-efficacy and enjoyment increased. Meanwhile, studies investigating the effects of natural environments on stress-related brain mechanisms suggest that exposure to nature can decrease amygdala activity (60). This suggests that the outdoor setting of OFE might act synergistically with physical exertion to enhance individuals’ mental health (61). The multiple merits of OFE intervention for personal mental health make it a powerful adjunct to mental health prevention.

At the same time, the OFE interventions improve psychosocial well-being, including reducing loneliness and enhancing interpersonal and communicative interactions. The meta-analysis results show a small positive effect of the OFE intervention on Loneliness (SMD = 0.33). This can be attributed to the fact that the OFE intervention increases opportunities for social engagement and the provision of social support. Previous studies confirmed that social support was significantly negatively correlated with loneliness (62, 63). And people with high self-efficacy received more social support and recognition (64). The evidence presented provides a potential explanation for why OFE interventions may reduce feelings of loneliness. Overall, OFE offers a green space for residents to exercise, promoting mental relaxation and stress relief and a daily space for socialization to enhance social interactions and improve residents’ quality of life and well-being.

### 4.5 Health-related issues

The meta-analysis results show that the OFE intervention has a large positive effect on fear of falls (SMD = 1.01). Falls have emerged as a prevalent health issue among seniors across the globe, according to the WHO. Every year, roughly 20–35% of people aged 62 and above suffer from falling incidents, and over 50% of these cases lead to injuries such as fractures, bleeding, and even death (65). Falls seriously impact patients’ physical and mental health and increase the healthcare burden on society. Healthcare expenditures increase with the frequency and severity of fall-related injuries (66). Falls risk factors include loss of balance, decreased muscle strength, and unsteady gait caused by reduced neurological control, which are primary triggers among older adults. Walking is the primary form of exercise for older adults, but it is ineffective in improving balance and strength and preventing falls. Combined exercise improved balance and muscle strength in older adults (67). This study’s results align with this finding. OFE interventions offer structured exercises that include various forms of exercise via diverse equipment, resulting in notable advancements in balance (SMD = 0.83) and lower body strength (SMD = 0.35). In this way, the OFE intervention can assist in preventing falls among older adults and promoting healthy aging.

The meta-analysis results indicate that the OFE intervention positively affects blood pressure, blood sugar, and lipids. There is substantial evidence showing that aerobic exercise may decrease blood pressure (68) and blood glucose levels (69), improve blood lipid profile (70), and lower the incidence of various diseases. Moreover, a study by the American Medical Association study found that moderate-intensity aerobic exercise has a practical antihypertensive effect with a maximum safety margin (71). It is more effective than low-intensity and vigorous-intensity exercise in preventing and reducing blood pressure. Studies, including this one, have found that the OFE intervention mainly involves moderate-intensity physical activity. Chow et al. employed a portable metabolic system to quantify the intensity of physical activity in older adults who used OFE (72). The range of metabolic equivalents for the air walkers was 2.81 to 3.55 METs, while the range for the ski machine was 3.02 to 4.05 METs. Studies have also measured metabolic equivalents, heart rate, and oxygen uptake during aerobic combined with resistance exercise with OFE. Results indicate that during bodyweight exercise, subjects had a mean metabolic equivalent of 4.6 METs, a mean heart rate of 64.1% of their maximum heart rate, and a mean oxygen uptake of 51.5% (73). Thus, the effectiveness of the OFE intervention in preventing and reducing blood pressure cannot be ignored.

### 4.6 Study limitations

This systematic review substantiates the effectiveness of OFE intervention on health outcomes but has several limitations. Firstly, the strength of the evidence is constrained by methodological limitations. Some included studies were non-RCTs, and nine RCTs contained a high or unclear risk of bias in certain domains. Furthermore, due to the limited number of studies included for each outcome (*n* < 10), we could not reliably assess publication bias using funnel plots or Egger’s tests. Consequently, the potential for small-study effects cannot be ruled out, and the reported aggregate effect sizes might be subject to overestimation. Secondly, the predominance of older female participants and the reliance on aggregated data from mixed-sex cohorts prevented the execution of stratified analyses by age or sex. Consequently, it is imperative to exercise caution when generalizing these findings to male populations or younger adults. Thirdly, using multiple assessment tools for a single outcome indicator in studies may lead to potentially bias effect size estimations, which complicate result interpretation in meta-analysis. Fourthly, the diversity of OFE results in various intervention program designs, making subgroup analyses and meta-regressions difficult, which do not allow for synthesizing specific exercise recommendations. Finally, due to the wide variety of assessment tools used across the included studies, it was not feasible to convert the standardized mean differences back to original units. Future research should prioritize standardized outcome measures to facilitate clearer clinical guidelines.

## 5 Conclusion

This systematic review and meta-analysis provide a comprehensive overview of the effectiveness of OFE intervention on health outcomes. The evidence indicates that OFE intervention effectively improves physical activity, physical fitness, psychological well-being, and other health-related results. However, due to the considerable variation in intervention characteristics and outcome measurements, it is not possible to determine which interventions are most beneficial. High-quality RCTs with well-reported OFE intervention characteristics are required to determine effective OFE exercise protocols and facilitate translation into practice.

## Data Availability

The original contributions presented in the study are included in the article/Supplementary material, further inquiries can be directed to the corresponding author.
