# Supplementary figures and images for: Effectiveness of outdoor fitness equipment intervention on health outcomes: a systematic review and meta-analysis
Source: Front Public Health. 2026 Feb 23;14:1701136. doi: 10.3389/fpubh.2026.1701136 (PMC12969065; doi:10.3389/fpubh.2026.1701136)

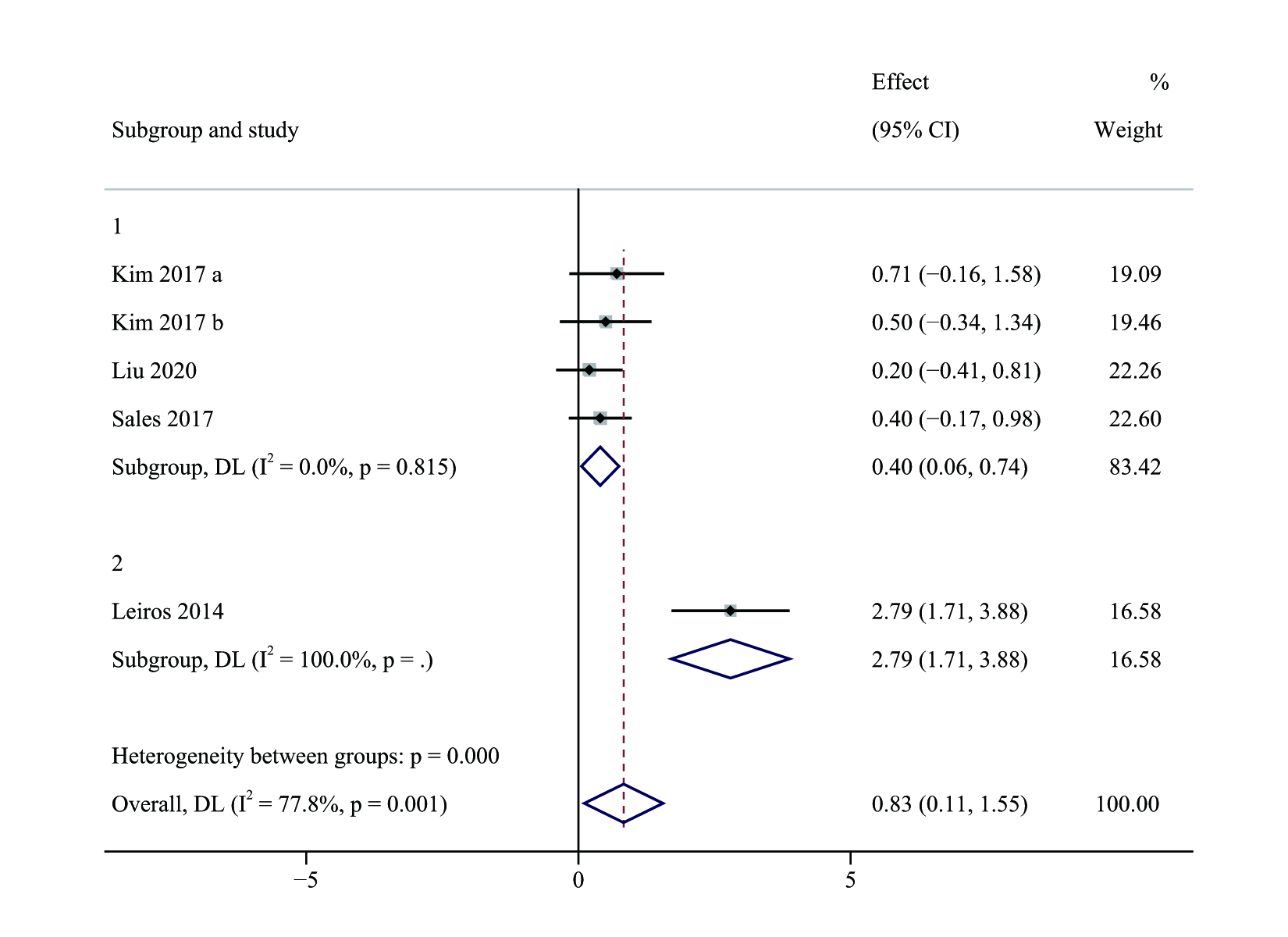

Supplement: Supplementary file 1 [file Image_1.TIF]

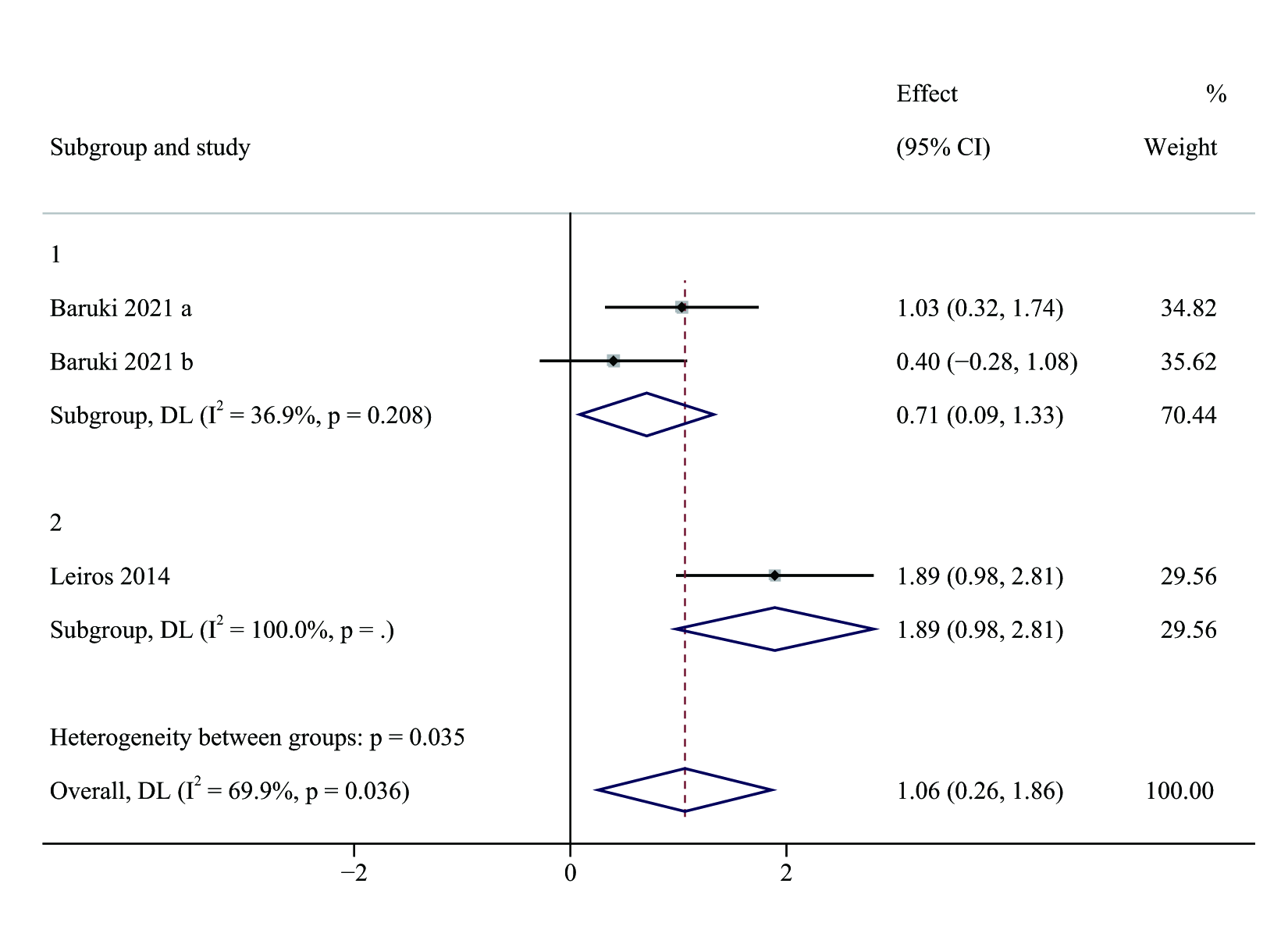

Supplement: Supplementary file 2 [file Image_2.TIF]
