# Supplementary material for: Effectiveness of outdoor fitness equipment intervention on health outcomes: a systematic review and meta-analysis
Source: Front Public Health. 2026 Feb 23;14:1701136. doi: 10.3389/fpubh.2026.1701136 (PMC12969065; doi:10.3389/fpubh.2026.1701136)
Supplement: Supplementary file 6 [file Table_1.DOCX]

（“exercis* space” OR “exercis* park” OR “outdoor exercis*” OR “outdoor recreation* area*” OR “fitness zone*” OR “outdoor* gym*” OR “outdoor fitness” OR “outdoor* park*” OR “outdoor recreation* facilit*” OR “public fitness facilit*” OR “recreation* park*” OR “communit* park*” OR “active park*” OR “bio-healthy park*” OR “healthy park*” OR “geriatric park*” OR “neighborhood park” OR “stretch station” OR “open gym*” OR “open air gym*” OR “fitness equipment”） AND （effic* OR evaluat* OR assess* OR measur* OR effect* OR validation OR indicator OR impact* OR benefi* OR screening OR risk OR outcome）
