# Supplementary material for: Effectiveness of outdoor fitness equipment intervention on health outcomes: a systematic review and meta-analysis
Source: Front Public Health. 2026 Feb 23;14:1701136. doi: 10.3389/fpubh.2026.1701136 (PMC12969065; doi:10.3389/fpubh.2026.1701136)
Supplement: Supplementary file 7 [file Table_2.DOCX]

Table 1 GRADE rates quality of evidence as follows

| Level | Explanation |
| --- | --- |
| High | There is a lot of confidence that the true effect lies close to that of the estimated effect. |
| Moderate | There is moderate confidence in the estimated effect: The true effect is likely to be close to the estimated effect, but there is a possibility that it is substantially different. |
| Low | There is limited confidence in the estimated effect: The true effect might be substantially different from the estimated effect. |
| Very low | There is very little confidence in the estimated effect: The true effect is likely to be substantially different from the estimated effect. |

Table 2 Factors influencing GRADE scores

| Factor Type | Factors | Explanation |
| --- | --- | --- |
| Downgrade | Risk of Bias | Studies with significant methodological flaws can decrease confidence in the effect estimate. |
|  | Inconsistency | Substantial unexplained heterogeneity or variability among the study results. |
|  | Indirectness | Evidence is not directly applicable to the population, intervention, comparator, or outcome of interest. |
|  | Imprecision | Wide confidence intervals or sparse data which make the effect estimate uncertain. |
|  | Publication Bias | Selective reporting of studies or outcomes may distort the true effect estimate. |
| Upgrade | Strong Association | The large magnitude of effect with no plausible confounders. |
|  | Dose-response gradient | The clear gradient of increased effect with increased dose or exposure. |
|  | All plausible confounding | Confounders would decrease the demonstrated effect or suggest a spurious effect when results show no effect. |
