# Supplementary material for: Effectiveness of outdoor fitness equipment intervention on health outcomes: a systematic review and meta-analysis
Source: Front Public Health. 2026 Feb 23;14:1701136. doi: 10.3389/fpubh.2026.1701136 (PMC12969065; doi:10.3389/fpubh.2026.1701136)
Supplement: Supplementary file 8 [file Table_3.docx]

Terminology variations and location of Outdoor Fitness Equipment

| First author, year | Terminology; locations |
| --- | --- |
| Barbosa et al.; 2024 | “outdoor gym equipment”; Public square |
| Baruki et al.; 2021 | “Outdoor fitness gym”; Public parks |
| Chow et al.; 2021 | “Outdoor fitness equipment”; Public parks |
| Johnson et al.; 2019 | “Outdoor gym”; city parks |
| Kim et al.; 2017 | “Outdoor exercise equipment”; public parks |
| Lee et al.; 2021 | “Outdoor exercise facilities”; community open spaces |
| Leiros-Rodríguez et al.; 2014 | “Equipment of a public park”; Public parks |
| Liu et al.; 2020 | “Outdoor fitness equipment”; Public parks |
| Levinger et al.; 2020 | “Seniors Exercise Park”; Public parks |
| Marcos-Pardo et al.; 2024 | "Outdoor Fitness Equipment"; Public square |
| Ng et al.; 2022 | “Seniors Exercise Park”; Public parks |
| Nguyen et al.; 2014 | “Fitness Zone Equipment”; Public parks |
| Plotnikoff et al.; 2023 | “Outdoor gym equipment”; Public parks |
| Sales et al.; 2017 | “Seniors Exercise Park”; Public parks |
