# Supplementary material for: Effectiveness of outdoor fitness equipment intervention on health outcomes: a systematic review and meta-analysis
Source: Front Public Health. 2026 Feb 23;14:1701136. doi: 10.3389/fpubh.2026.1701136 (PMC12969065; doi:10.3389/fpubh.2026.1701136)
Supplement: Supplementary file 9 [file Table_4.docx]

Characteristics of the equipment used in included studies

| **First author, year** | **Number of facilities** | **Name of Facility** | **Function** | **Adaptability** | **Progression** |
| --- | --- | --- | --- | --- | --- |
| Baruki et al.; 2021 | 4 | Elliptical; Rowing; Rambler; Leg Press | Improves cardiovascular endurance, balance, coordination, and lower-body muscle strength. | NO | Yes (Volume, Speed, Rest; HR/RPE - guided) |
| Barbosa et al.; 2024 | 7 | Air walker; Ski machine; Multi exerciser; Rower; Shoulder wheel; Double surf board ; Leg press | Improves cardiorespiratory endurance, muscular strength, flexibility, and functional capacity. | NO | NO |
| Chow et al.; 2021 | 7 | Air walker; Ski machine; Rowing machine; Bonny rider; Arm stretch; Shoulder wheel; Waist twister | Improves cardiorespiratory fitness, muscular strength, flexibility, and balance. | NO | Yes (Resistance/Balance: Reps) |
| Johnson et al.; 2019; Sweden | 6 | NR | NR | NO | NO |
| Kim et al.; 2017; Korea ^b^ | 5 | pull weight; chair pull; leg extension; sky-walk; cross country | Enhances cardiovascular endurance, muscular strength, and physical function in the upper and lower body. | NO | Yes (Increasing RPE targets) |
| Lee et al.; 2021; China | NR | NR | NR | NO | NO |
| Leiros-Rodríguez et al.; 2014; Spain ^b^ | 12 | NR | Improves static and dynamic balance required for daily functionality. | NO | Yes (Task complexity: Base of support) |
| Liu et al.; 2020; China ^b^ | 3 | Air walker; arm stretcher; leg press | Targets cardiorespiratory function, flexibility, and muscle strength. | NO | Yes (Duration, Sets, Reps) |
| Levinger et al.; 2020; Austria ^b^ | 22 | Seniors Exercise Park | Improves strength, balance, joint flexibility, and mobility. | NO | Yes (Exercise duration & Rest) |
| Marcos-Pardo et al.; 2024; Spain ^b^ | 8 | Bonny rider; Air Walker; Surfboard; Row; Parallel bars; Gemini; Flyer wheels; Swing | Improves muscular strength, cardiovascular endurance, flexibility, and functional capacity. | NO | Yes (Duration, Reps, Rest) |
| Ng et al.; 2022; Austria ^b^ | 22 | Seniors Exercise Park: Pull-ups; Calf raises and finger steps; Gangway Hand roll; Ramp and net, Snake pipe-big wave; Sit to stand; Stairs; Hip extension; Push-ups; Shoulder arches; Balance stool; Balance beam; Core twister; Snake pipe- small wave; Step ups; Taps on platform; Hip abduction | Improves balance, strength, flexibility, coordination, and cardiovascular endurance. | YES | Yes (Performance-based) |
| Nguyen et al.; 2014; USA | 6 | Leg Press; Unilateral Leg Extension; Chest Press;  Lat-pull down; Vertical Press; Elliptical | Enhances full-body muscular endurance and cardiorespiratory health. | NO | Yes (Aerobic: Duration; Resistance: Sets/Reps) |
| Plotnikoff et al.; 2023; Austria ^b^ | There are 12 parks in total, each containing between 3 and 19 pieces of equipment. | Step-up; push-up; sit-up; chin-up; shoulder press et al. | Increases muscular strength, cardiovascular health, and functional fitness. | NO | Yes（Self-selected difficulty） |
| Sales et al.; 2017; Austria ^b^ | 16 | Push-ups; Modified pull-ups; Balance stool; Sit to stand; Ramp + Net + Climb through; Balance beam; Steps; Step-ups; Taps on platform; Gangway; Calf raises + Finger steps; Round snake pipe; Sharp snake pipe; Hip extension; Screws/turners; Hip abduction | Enhances functional fitness, mobility, fine motor skills, balance, coordination, strength, and flexibility. | NO | NO |
