# Supplementary material for: Effectiveness of outdoor fitness equipment intervention on health outcomes: a systematic review and meta-analysis
Source: Front Public Health. 2026 Feb 23;14:1701136. doi: 10.3389/fpubh.2026.1701136 (PMC12969065; doi:10.3389/fpubh.2026.1701136)
Supplement: Supplementary file 10 [file Table_5.docx]

|  | Baruki et al. 2021 | Barbosa et al.; 2024 | Chow et al. 2021 | Johnson et al. 2019 | Kim et al. 2017 | Lee et al. 2021 | Leiros et al. 2014 | Levinger et al. 2020 | Liu et al. 2020 | Marcos-Pardo et al.; 2024 | Ng et al. 2022 | Nguyen et al. 2014 | Plotnikoff et al. 2023 | Sales et al. 2017 |
| --- | --- | --- | --- | --- | --- | --- | --- | --- | --- | --- | --- | --- | --- | --- |
| **Reporting** |  |  |  |  |  |  |  |  |  |  |  |  |  |  |
| Q1 Hypothesis/aim/objective clearly described | 1 | 1 | 1 | 1 | 1 | 1 | 1 | 1 | 1 | 1 | 1 | 1 | 1 | 1 |
| Q2 Main outcomes in Introduction or Methods | 1 | 1 | 1 | 1 | 1 | 1 | 1 | 1 | 1 | 1 | 1 | 1 | 1 | 1 |
| Q3 Patient characteristics clearly described | 1 | 1 | 1 | 1 | 1 | 1 | 1 | 1 | 1 | 1 | 1 | 1 | 1 | 1 |
| Q4 Interventions of interest clearly described | 1 | 1 | 1 | 1 | 1 | 1 | 1 | 1 | 1 | 1 | 1 | 1 | 1 | 1 |
| Q5 Principal confounders clearly described | 1 | 1 | 1 | 1 | 1 | 1 | 1 | 1 | 1 | 1 | 1 | 1 | 1 | 1 |
| Q6 Main findings clearly described | 1 | 1 | 1 | 1 | 1 | 1 | 1 | 1 | 1 | 1 | 1 | 1 | 1 | 1 |
| Q7 Estimates of random variability provided for main outcomes | 1 | 1 | 1 | 1 | 1 | 0 | 1 | 1 | 1 | 1 | 1 | 1 | 1 | 1 |
| Q8 All adverse events of intervention reported | 0 | 0 | 0 | 0 | 0 | 0 | 0 | 0 | 0 | 0 | 0 | 0 | 0 | 0 |
| Q9 Characteristics of patients lost to follow-up described | 1 | 1 | 1 | 1 | 1 | 1 | 1 | 1 | 1 | 1 | 1 | 1 | 1 | 1 |
| Q10 Probability values reported for main outcomes | 1 | 1 | 1 | 1 | 1 | 1 | 1 | 1 | 1 | 1 | 1 | 1 | 1 | 1 |
| **External validity** |  |  |  |  |  |  |  |  |  |  |  |  |  |  |
| Q11 Subjects asked to participate were representative of source population | UTD | UTD | UTD | UTD | UTD | UTD | UTD | UTD | UTD | UTD | UTD | UTD | UTD | UTD |
| Q12 Subjects prepared to participate were representative of source population | UTD | UTD | UTD | UTD | UTD | UTD | UTD | UTD | UTD | UTD | UTD | UTD | UTD | UTD |
| Q13 Location and delivery of study treatment was representative of source population | 1 | 1 | 1 | 1 | 1 | 1 | 1 | 1 | 1 | 1 | 1 | 1 | 1 | 1 |
| **Internal validity - bias** |  |  |  |  |  |  |  |  |  |  |  |  |  |  |
| Q14 Study participants blinded to treatment | 1 | 1 | 0 | 0 | 0 | 0 | 0 | 0 | 1 | 1 | 0 | 0 | 0 | 1 |
| Q15 Blinded outcome assessment | 1 | 0 | 0 | 0 | 1 | 0 | 0 | 0 | 1 | 0 | 0 | 0 | 0 | 1 |
| Q16 Any data dredging clearly described | 1 | 1 | 1 | 1 | 1 | 1 | 1 | 1 | 1 | 1 | 1 | 1 | 1 | 1 |
| Q17 Analyses adjust for differing lengths of follow-up | 1 | 1 | 1 | 1 | 1 | 1 | 1 | 1 | 1 | 1 | 1 | 1 | 1 | 1 |
| Q18 Appropriate statistical tests performed | 1 | 1 | 1 | 1 | 1 | 1 | 1 | 1 | 1 | 1 | 1 | 1 | 1 | 1 |
| Q19 Compliance with interventions was reliable | 1 | 1 | 1 | 1 | 1 | 1 | 1 | 1 | 1 | 1 | 1 | 1 | 1 | 1 |
| Q20 Outcome measures were reliable and valid | 1 | 1 | 1 | 1 | 1 | 1 | 1 | 1 | 1 | 1 | 1 | 1 | 1 | 1 |
| **Internal validity -confounding** |  |  |  |  |  |  |  |  |  |  |  |  |  |  |
| Q21 All participants recruited from the same source population | 1 | 1 | 1 | 1 | 1 | 1 | 1 | 1 | 1 | 1 | 1 | 1 | 1 | 1 |
| Q22 All participants recruited over the same time period | 1 | 1 | 1 | 1 | 1 | 1 | 1 | 1 | 1 | 1 | 1 | 1 | 1 | 1 |
| Q23 Participants randomized to treatment(s) | 1 | 1 | 0 | 0 | 1 | 0 | 1 | 0 | 1 | 1 | 0 | 0 | 1 | 1 |
| Q24 Allocation of treatment concealed from investigators and participants | 1 | 1 | 0 | 0 | 0 | 0 | 0 | 0 | 1 | 1 | 0 | 0 | 0 | 1 |
| Q25 Adequate adjustment for confounding | 1 | 1 | 1 | 1 | 1 | 1 | 1 | 1 | 1 | 1 | 1 | 1 | 1 | 1 |
| Q26 Losses to follow-up taken into account | 1 | 1 | 1 | 1 | 1 | 1 | 1 | 1 | 1 | 1 | 1 | 1 | 1 | 1 |
| **Power** |  |  |  |  |  |  |  |  |  |  |  |  |  |  |
| Q27 Sufficient power to detect treatment effect at significance level of 0.05 | 1 | 1 | 1 | 0 | 1 | 1 | 1 | 1 | 1 | 1 | 1 | 0 | 1 | 1 |
| **Total score** | 24 | 23 | 20 | 19 | 22 | 19 | 21 | 20 | 24 | 23 | 20 | 19 | 21 | 24 |
