# Supplementary material for: Effectiveness of outdoor fitness equipment intervention on health outcomes: a systematic review and meta-analysis
Source: Front Public Health. 2026 Feb 23;14:1701136. doi: 10.3389/fpubh.2026.1701136 (PMC12969065; doi:10.3389/fpubh.2026.1701136)
Supplement: Supplementary file 11 [file Table_6.DOCX]

| **Certainty assessment** | | | | | | | **№ of patients** | | **Effect** | | **Certainty** | **Importance** |
| --- | --- | --- | --- | --- | --- | --- | --- | --- | --- | --- | --- | --- |
| **№ of studies** | **Study design** | **Risk of bias** | **Inconsistency** | **Indirectness** | **Imprecision** | **Other considerations** | **Intervention** | **Comparison** | **Relative (95% CI)** | **Absolute (95% CI)** |  |  |
| **Cardiorespiratory fitness** | | | | | | | | | | | | |
| 4 | randomized trials | not serious | not serious | not serious | not serious | none | 106 | 98 | - | SMD **0.53 SD higher** (0.25 higher to 0.82 higher) | ⨁⨁⨁⨁ High | CRITICAL |
| **Lower limb muscle strength** | | | | | | | | | | | | |
| 5 | randomized trials | serious | not serious | not serious | not serious | none | 228 | 221 | - | SMD **0.32 SD higher** (0.13 higher to 0.51 higher) | ⨁⨁⨁◯ Moderate | IMPORTANT |
| **Upper limb muscle strength** | | | | | | | | | | | | |
| 5 | randomized trials | serious | not serious | not serious | not serious | none | 228 | 221 | - | SMD **0.25 SD higher** (0.06 higher to 0.44 higher) | ⨁⨁⨁◯ Moderate | IMPORTANT |
| **Balance** | | | | | | | | | | | | |
| 4 | randomized trials | serious | not serious | not serious | not serious | strong association | 87 | 76 | - | SMD **0.83 SD higher** (0.11 higher to 1.55 higher) | ⨁⨁⨁⨁ High | CRITICAL |
| **Quality of life** | | | | | | | | | | | | |
| 2 | randomized trials | serious | serious | not serious | not serious | strong association | 47 | 50 | - | SMD **1.06 SD higher** (0.26 higher to 1.86 higher) | ⨁⨁⨁◯ Moderate |  |

**CI:** confidence interval; **SMD:** standardized mean difference
